# Effectiveness of Electric Muscle Stimulation (EMS) and Transcutaneous Electric Nerve Stimulation (TENS) in patients with overactive bladder: A randomized controlled trial

**DOI:** 10.12669/pjms.41.11.12777

**Published:** 2025-11

**Authors:** Sajid Rashid, Omama Sajid

**Affiliations:** 1 Sajid Rashid, Multan Medical & Dental College, Multan, Pakistan; 2 Omama Sajid, Multan Medical & Dental College, Multan, Pakistan

**Keywords:** Nerve stimulation, Overactive bladder, Transcutaneous electric nerve stimulation, Urinary Incontinence

## Abstract

**Objective:**

To compare the outcome of the patients of Overactive Bladder with traditional treatment alone and with EMS and TENS in study groups.

**Methodology:**

A randomized controlled trial was conducted at Ibn e Siena Hospital, Multan, spanned from October, 2019 to March, 2021 with IRB approval (Letter No. C-32-120, dated January 31, 2020). A total of 315 patients aged 35-60 years with overactive bladder symptoms were stratified into three groups i.e., Control, EMS and TENS (n=105 each). The control group received anticholinergic drugs and pelvic floor exercises, while the TENS and EMS groups received respective modalities along with conventional treatment for the duration of 12 weeks. Symptom severity was assessed using the Overactive Bladder Symptom Score (OABSS). One-way ANOVA and post hoc analysis were used for statistical comparison.

**Results:**

All three treatments reduced symptom severity however EMS showed the highest improvement (20%), followed by TENS (9.5%) and then Control (7.6%). EMS and TENS groups showed statistically significant improvements compared to the control.

**Conclusion:**

Neuromodulation offers a promising, noninvasive approach for managing overactive bladder (OAB). This study demonstrated that both EMS and TENS significantly reduced OAB symptoms, with EMS showing superior efficacy across all parameters. These findings support EMS as a safe and effective treatment option, particularly for patients seeking alternatives to pharmacologic or surgical interventions.

## INTRODUCTION

Overactive Bladder (OAB) syndrome is defined by the International Continence Society (ICS) as a condition characterized by urinary urgency, typically accompanied by increased daytime frequency, nocturia and sometimes urge urinary incontinence (UUI), in the absence of urinary tract infection or other identifiable pathology.^1^ OAB affects individuals across all age groups, but prevalence increases significantly with age. This condition negatively impacts quality of life, affecting sleep, sexual health, mental well-being and occupational productivity and may lead to social isolation.^2^

The urinary bladder is the key organ involved in micturition, which occurs in two main phases: urine storage and emptying. These phases are regulated by coordinated interactions between the bladder, pelvic floor muscles and the central and peripheral nervous systems.^3^ During the storage phase, the detrusor muscle relaxes while the urinary sphincters contract; during voiding, the bladder contracts while the sphincters relax, allowing urine to be expelled. In OAB, even when the bladder is not full, the control mechanisms malfunction, leading to involuntary detrusor muscle contractions, resulting in a perceived loss of voluntary bladder control.^4^

The hallmark urodynamic feature of OAB is detrusor overactivity, which refers to involuntary detrusor contractions during the bladder filling phase. These contractions may occur spontaneously or be provoked. In men, OAB is often associated with detrusor overactivity. Epidemiological studies estimate that up to 36% of adult women in Europe and the U.S. experience OAB symptoms, highlighting its significant global burden.^5^

Transcutaneous Electrical Nerve Stimulation (TENS) inhibits the micturition reflex by activating dermatome nerve fibers, which suppress sensory signals from the bladder based on the pain gate theory. It acts on the bladder, sphincter, and pelvic floor to reduce detrusor hyperexcitability by modulating preganglionic neurons involved in the reflex. Electrical Muscle Stimulation (EMS) enhances bladder capacity by exciting pelvic floor nerves, thereby restricting bladder emptying through spinal reflexes. This mechanism is linked to pelvic-spinal touch reflexes, potentially evolved to control urination during intercourse. Both TENS and EMS demonstrate promising roles in managing urinary incontinence and overactive bladder.^6^-^7^

This study aimed to see the effectiveness of EMS and TENS as non-invasive and cost-effective treatment options for managing patients with overactive bladder in Pakistan. By reducing urgency, frequency, and dribbling, neuromodulation can enhance physical functioning, independence, and quality of life. The findings will also provide physiotherapists with evidence-based guidance for incorporating neuromodulation into clinical practice for the management of OAB.

## METHODOLOGY

This randomized controlled trial was aimed to compare the effectiveness of Transcutaneous Electrical Nerve Stimulation (TENS) and Electrical Muscle Stimulation (EMS) in managing Overactive Bladder (OAB). This trial was conducted at Ibn e Siena Hospital and Research Institute, Multan. The study spanned from October 2019 to March 2021.

### Ethical Approval:

Ethical approval was granted by the Advanced Studies and Research Committee (ASRC) of Isra Institute of Rehabilitation Sciences, Isra University, Islamabad (Letter No. C-32-120; Dated: January 31, 2020) and the trial was registered at Clinical Trials.gov (Registry No: NCT04364438). Informed written consent was obtained from all participants. The Sample size was based on detecting between-group differences in OABSS using one-way ANOVA. With α = 0.05 and 80% power, the total sample of 315 patients (105 per group) provides power to detect an effect size of f ≈ 0.158 (≈Cohen’s d 0.32), equivalent to an absolute difference of about 1–1.6 OABSS points depending on SD. TENS, EMS and Control using a computer-generated randomization sequence. Allocation was concealed using sequentially numbered, opaque, sealed envelopes prepared by an independent researcher not involved in enrolment or assessment. To reduce bias, outcome assessors were blinded to group allocation.

### Inclusion & Exclusion Criteria:

It included adults aged 35-60 years of both genders with OAB symptoms for more than three months and a frequency of more than eight urinations per 24 hours. Exclusion criteria included recent urinary tract infections (within four months), stress incontinence, pelvic organ prolapse, obstructive uropathy, prostatic hyperplasia, malignancy or unwillingness to consent.^8^ Participants were selected using simple random sampling based on age and gender to ensure homogeneity. The data normality was tested using the Shapiro-Wilk test. This test indicated that data in all three groups were normally distributed (TENS Group: p = 0.247, EMS Group: p = 0.312, Control Group: p = 0.278). After screening and obtaining consent, participants completed a baseline demographic and symptom questionnaire, including the Overactive Bladder Symptom Score (OABSS).

The questionnaire was administered in English and Urdu with researcher assistance for clarity. The TENS and EMS groups received electrical stimulation in addition to conventional treatment (anticholinergic medications, bladder training, pelvic floor exercises and antibiotics). Interventions were delivered via four self-adhesive electrodes placed over the sacral region (S2-S3). For the TENS group, stimulation was delivered using surface electrodes placed over sacral dermatomes S2-S3 with parameters set at a frequency of 50 Hz, pulse width 200 μs, continuous mode, and intensity adjusted to a strong but comfortable sensory level (below motor threshold) by using TENS ES-320, ITO, Japan.^9^ For the EMS group, surface electrodes were used to stimulate the pelvic floor muscles with a frequency of 100–150 Hz, pulse width 300 μs, duty cycle of 2:1 (10 seconds on, five seconds off), and amplitude increased until a visible contraction was observed without discomfort by using EMS Trio 300, ITO, Japan.^10^ Each session lasted 20 minutes, five times a week for 12 weeks. Stimulation intensity was gradually increased to a level just below discomfort. The control group received only conventional pharmacological therapy without any electrical stimulation. The primary outcome was the change in OAB symptoms, measured via OABSS at baseline and post-intervention. All post-treatment assessments followed the same protocol as baseline. Data were analyzed using SPSS version 21. Descriptive statistics (mean ± SD) were calculated and one-way ANOVA followed by post hoc tests was used for intergroup comparisons. A p-value < 0.05 was considered statistically significant.

## RESULTS

A total of 315 participants were enrolled and equally divided into three groups: TENS (n=105), EMS (n=105) and Control (n=105). The effectiveness of interventions was assessed by comparing pre- and post-treatment frequencies of OAB symptoms including daytime frequency, night-time frequency, urgency and dribbling, as well as symptom severity based on Overactive Bladder Symptom Score (OABSS). The age distribution across all groups was similar, indicating effective randomization. The mean ages in Control (48.52±6.9), EMS (47.79±7.4) and TENS (48.43±6.9) groups were statistically comparable, ensuring baseline uniformity. The BMI analysis showed that the majority of participants were overweight in all three groups, with small variations. The EMS group had the highest percentage of healthy-weight individuals (43.8%), while the TENS group had the highest overweight percentage (68.6%). These findings suggest that BMI distribution was comparable with no significant baseline discrepancy.

**Fig.1:**
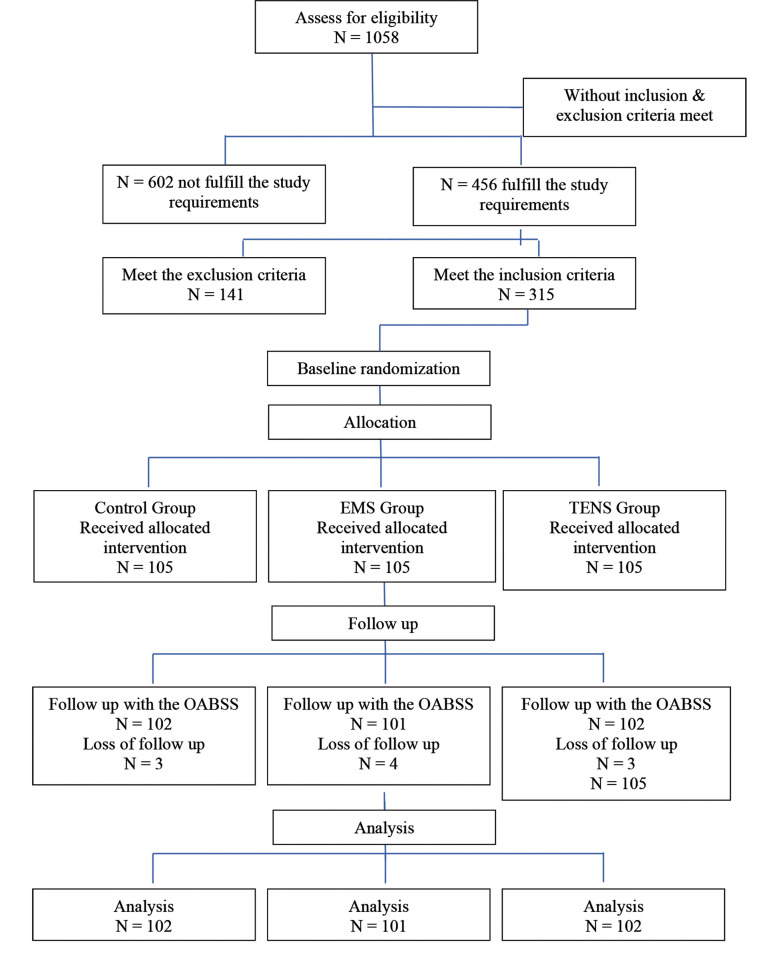
Consort flow diagram.

The pre- and post-treatment comparison within groups exhibited reduction in all four symptoms i.e. Daytime frequency, Night time frequency, Urgency and dribbling across the three groups, but EMS showed the greatest improvement across all parameters. EMS led to the highest reduction in night-time frequency (25.7%), followed by daytime frequency (18.1%), urgency (16.2%) and dribbling (13.4%), while TENS demonstrated reduction in night-time frequency (15.3%), daytime frequency (10.5%), urgency (12.4%) and dribbling (2.9%). The control group showed reduction in night-time frequency (11.4%), daytime frequency (13%), urgency (9.5%) and dribbling (13%).

OAB symptom severity was categorized into mild, moderate, severe and normal based on OABSS scores. OABSS category shifts confirmed the clinical efficacy of EMS, with the highest transition to the ‘normal’ category (20%), while TENS yielded 9.5% and the control group achieved 7.6%. EMS was also most effective in reducing moderate and severe symptom categories.

A post hoc one-way ANOVA was conducted to assess the statistical significance between group outcomes based on OABSS. This analysis using Post Hoc ANOVA confirmed that EMS significantly outperformed both TENS (p = .026) and Control (p = .003) in reducing OAB symptoms based on OABSS scores. Although TENS showed clinical benefits, its difference from the control group was not statistically significant (p = .738). The mean difference between EMS and TENS groups was 1.238 (p = 0.026), while the mean difference between EMS and Control was 1.590 (p = 0.003), favoring EMS treatment for greater symptom reduction.

Post-hoc analysis using Tukey’s HSD test revealed that EMS produced significantly greater improvements compared to the Control group (mean difference = 0.317, p = 0.030). The difference between TENS and EMS approached significance but did not reach the 0.05 threshold (mean difference = -0.288, p = 0.056). There was no significant difference between TENS and Control (p = 0.970).

## DISCUSSION

Overactive bladder (OAB) is commonly managed with pharmacologic agents, bladder training or surgery. However, anticholinergic medications are associated with adverse effects that limit adherence, bladder training requires time and motivation and surgery carries risks, making it a less preferred option. These limitations have led to growing interest in noninvasive neuromodulation techniques such as Electrical Stimulation (ES), including Transcutaneous Electrical Nerve Stimulation (TENS) and Electrical Muscle Stimulation (EMS), for managing lower urinary tract dysfunction.^11^,^12^

Our study explored stimulation of alternative pathways such as S2-S3 dermatomes via TENS and pelvic floor muscles with EMS. The results of our study indicate that both EMS and TENS can activate these nerves noninvasively, with EMS showing a notably higher clinical efficacy. Specifically, EMS led to a statistically significant improvement in OABSS score potentially enhancing therapeutic effectiveness. Previous researches predominantly focused on PTNS and TTNS with mixed outcomes. Preclinical investigations have shown that these nerves possess inhibitory effects on bladder activity, with SAFN stimulation demonstrating superior efficiency at lower amplitudes compared to tibial nerve stimulation.^13^-^15^

**Table-I:** Comparison of Frequency of Incontinence Symptoms.

Frequency incontinence				Day time frequency	Night time frequency	Urgency	Dribbling
Control	Pre	Frequency %	Yes	80(77.1%)	92(87.6%)	75(71.4%)	55(52.4%)
			No	25(22.9%)	13(12.4%)	30(28.6%)	47(44.8%)
	Post	Frequency %	Yes	68(64.8%)	80(76.2%)	65(61.9%)	41(39%)
			No	34(32.4%)	22(21%)	37(35.2%)	64(60%)
	Percentage Improvement			13%	11.4%	9.5%	13%
EMS	Pre	Frequency %	Yes	80(76.2%)	91(86.7%)	65(61.9%)	43(41%)
			No	25(23.8%)	14(13.3%)	40(38.1%)	61(58.1%)
	Post	Frequency %	Yes	61(58.1%)	64(61%)	48(45.7%)	29(27.6%)
			No	40(38.1%)	37(35.2%)	53(50.5%)	72(68.8%)
	Percentage Improvement			18.1%	25.7%	16.2%	13.4%
TENS	Pre	Frequency %	Yes	84(80%)	93(88.6%)	66(62.9%)	40(38.1%)
			No	21(20%)	12(11.4%)	39(37.1%)	65(61.9%)
	Post	Frequency %	Yes	73(69.5%)	77(73.3%)	53(50.5%)	37(35.2%)
			No	29(27.6%)	25(23.8%)	49(46.7%)	66(62.9%)
	Percentage Improvement			10.5%	15.3%	12.4%	2.9%

**Table-II:** Comparison of OABSS Severity Categories.

OABSS			Mild	Moderate	Severe	Normal
Control	Pre	Frequency %	22(21%)	66(62.9%)	17(16.2%)	0(0%)
	Post	Frequency %	27(25.7%)	58(55.2%)	9(8.6%)	8(7.6%)
	Percentage Change		4.7% Increase	7.7 % Decrease	7.6 % Decrease	7.6% Increase
EMS	Pre	Frequency %	22(21%)	69(65.7%)	14(13.3%)	0(0%)
	Post	Frequency %	38(36.2%)	36(34.3%)	6(5.7%)	21(20%)
	Percentage Change		15.2% Increase	31.4% Decrease	7.6 % Decrease	20% Increase
TENS	Pre	Frequency %	24(21.9%)	66(62.9%)	15(14.3%)	0(0%)
	Post	Frequency %	27(25.7%)	54(51.4%)	11(10.5%)	10(9.5%)
	Percentage Change		3.8% Increase	11.5 % Decrease	3.8 % Decrease	9.5% Increase

The current findings also support a wider recognition of electrical stimulation as a valid, non-pharmacological intervention for urinary incontinence. In our study, EMS facilitated the greatest symptom category shift in the Overactive Bladder Symptom Score (OABSS), with 20% of participants transitioning to the “normal” category and showing the most notable reductions in moderate and severe symptoms. TENS, although clinically beneficial in some cases, did not reach statistical significance compared to the control group (p = 0.738), whereas EMS yielded significantly better outcomes than both TENS (p = 0.026) and control (p = 0.003). Paker MK et al. have classified electrical stimulation as an essential component of pelvic floor muscle training (PFMT), reporting an approximate 26% reduction in incontinence episodes through conservative approaches.^16^

Comparative analysis of symptom-specific outcomes further highlights EMS’s superiority. Reductions in nighttime frequency (25.7%), daytime frequency (18.1%), and urgency episodes (16.2%) were highest in the EMS group. TENS produced moderate improvements, while the control group demonstrated minimal change. These improvements were mirrored by OABSS category shifts, underscoring EMS as the most effective modality among those tested. The results of our study are consistent with findings of Ghavidel-Sardsahra A et al. who conducted a systematic review and meta-analysis comparing TTNS and PTNS to control interventions. Both stimulation methods significantly reduced daily voiding frequency and nocturia and increased mean voided volume. These observations are in line with the current study, where EMS and TENS significantly reduced urgency, frequency, and dribbling episodes.^17^

**Table III:** Post-hoc Tukey’s HSD multiple comparisons for OABSS.

(I) Group	(J) Group	Mean Difference (I-J)	Std. Error	Sig.	95% Confidence Interval	
					Lower Bound	Upper Bound
TENS	EMS	-.28761	.12442	.056	-.5807	.0054
	Control	.02941	.12412	.970	-.2629	.3217
EMS	TENS	.28761	.12442	.056	-.0054	.5807
	Control	.31703*	.12442	.030	.0240	.6101
Control	TENS	-.02941	.12412	.970	-.3217	.2629
	EMS	-.31703*	.12442	.030	-.6101	-.0240

* The mean difference is significant at the 0.05 level.

The findings of our study corroborate the findings of Yildiz N et al. who conducted randomized controlled trial in women with idiopathic OAB and demonstrated that combining electrical stimulation with bladder training and biofeedback yielded the most favorable outcomes. Participants receiving electrical stimulation-either alone or in combination showed significantly greater improvements in symptom severity, voiding frequency, and treatment satisfaction.^18^

The present study helps bridge this gap by providing defined stimulation parameters for both EMS and TENS, contributing to a more standardized approach for future interventions. Our study adds to the growing body of evidence supporting noninvasive electrical stimulation as an effective strategy for OAB management. EMS, in particular, demonstrated superior performance across several clinical metrics, emphasizing its potential as a first-line conservative treatment option in patients with OAB, especially when pharmaceutical approaches are not suitable. Sayner AM et al. highlighted the variability in treatment parameters as a barrier to generalizability.^19^

### Strength of the study:

A key strength of the study was the ability of participants to self-administer therapy, reflecting a patient-centered care model. Compliance was high (306 out of 315 participants) and bladder diaries confirmed sustained symptom improvement over 12 weeks. In contrast to TTNS and PTNS studies, which mostly included mild OAB cases^4^,^15^, this study involved participants with moderate to severe symptoms (11.5-28.3 voids/day) and still showed significant benefit. The findings of current study suggest that noninvasive stimulation of sacral plexus and lower limb dermatomes is an effective alternative to invasive procedures. EMS and TENS provide safer, cost-effective and noninvasive alternatives.

### Limitations:

Despite positive outcomes, limitations include variability in stimulation settings, anatomical differences and adherence. Future larger-scale studies with longer follow-up, sham-controlled trials with more rigorous blinding are warranted to validate these findings and optimize protocols.

## CONCLUSION

Neuromodulation offers a promising, noninvasive approach for managing overactive bladder (OAB). This study demonstrated that both EMS and TENS significantly reduced OAB symptoms, with EMS showing trend towards superior efficacy across all parameters. These findings support EMS as a safe and effective treatment option, particularly for patients seeking alternatives to pharmacologic or surgical interventions.

## References

[1] Sheikh MA, Fawad A, Rabbani KJ, Mazhar SB, Ali S, Yasmin H (2022). Overactive bladder:A multicenter study in Pakistan. J Pak Med Assoc.

[2] Jensen S, Walker D, Elsouda D, Lockefeer A, Kenton K, Peipert JD (2024). An observational, patient reported outcome study of sleep quality and depression among individuals with overactive bladder syndrome. Neurourol Urodyn.

[3] Rashid S, Babur MN, Khan RR, Khalid MU, Mansha H, Riaz S (2021). Prevalence and associated risk factors among patients with overactive bladder syndrome in Pakistan. Pak J Med Sci.

[4] Aydogmus H, Sengul M, Bolel O, Kotan TS (2025). Effectiveness of transcutaneous tibial nerve stimulation on overactive bladder treatment. Pak J Med Sci.

[5] Nitti VW, Patel A, Karram M (2021). Diagnosis and management of overactive bladder:A review. J Obstet Gynaecol Res.

[6] Xavier B (2024). Optimization of electrical stimulation/modulation therapies in the treatment of neurogenic and non-neurogenic lower urinary tract dysfunctions. Diss Univ Antwerp.

[7] Wyndaele JJ (2022). Sensations in the Urinary Bladder. Sensation Pelvic Region.

[8] Filippini M, Biordi N, Curcio A, Comito A, Pennati BM, Farinelli M (2023). A qualitative and quantitative study to evaluate the effectiveness and safety of magnetic stimulation in women with urinary incontinence symptoms and pelvic floor disorders. Medicina.

[9] Amer-Cuenca JJ, Badenes-Ribera L, Biviá-Roig G, Arguisuelas MD, Suso-Martí L, Lisón JF (2023). The dose-dependent effects of transcutaneous electrical nerve stimulation for pain relief in individuals with fibromyalgia:a systematic review and meta-analysis. Pain.

[10] García-Aguirre R, Torres-Treviño L, Quiroz-Compean G, Rodríguez-Liñan A (2023). Development of a muscle electrical stimulation parameter selection method with an intelligent system. Eng Appl Artif Intell.

[11] Su JS, Mazeaud C, Khavari R (2023). Central Nervous Stimulation for Neurogenic Lower Urinary Tract Dysfunction:Current Application and Emergent Therapies. Curr Bladder Dysfunction Rep.

[12] Nishii H (2021). A review of aging and the lower urinary tract:the future of urology. Int Neurourol J.

[13] Mostafaei H, Mori K, Quhal F, Miura N, Motlagh RS, Pradere B (2021). Nocebo response in the pharmacological management of overactive bladder:a systematic review and meta-analysis. Eur Urol Focus.

[14] Liu P, Li Y, Shi B, Zhang Q, Guo H (2022). Comparison of different types of therapy for overactive bladder:A systematic review and network meta-analysis. Front Med.

[15] Booth J, Aucott L, Cotton S, Davis B, Fenocchi L, Goodman C (2021). Tibial nerve stimulation compared with sham to reduce incontinence in care home residents:ELECTRIC RCT. Health Technol Assess.

[16] Paker MK, Yuvaci HU, Kilic Y, Nas K, Bostanci MS, Cevrioglu AS (2023). Comparison of the effectiveness of pelvic floor muscle training, biofeedback, and tibial nerve stimulation in overactive bladder syndrome:A prospective randomized controlled study. J Clin Obstet Gynecol.

[17] Ghavidel-Sardsahra A, Ghojazadeh M, Rahnama'I MS, Naseri A, Yazdandoost S, Khezerloo T (2022). Efficacy of percutaneous and transcutaneous posterior tibial nerve stimulation on idiopathic overactive bladder and interstitial cystitis/painful bladder syndrome:a systematic review and meta-analysis. Neurourol Urodyn.

[18] Yildiz N, Alkan H, Sarsan A (2021). Efficacy of intravaginal electrical stimulation added to bladder training in women with idiopathic overactive bladder:A prospective randomized controlled trial. Int Braz J Urol.

[19] Sayner AM, Rogers F, Tran J, Jovanovic E, Henningham L, Nahon I (2022). Transcutaneous Tibial Nerve Stimulation in the Management of Overactive Bladder:A Scoping Review. Neuromodulation.

